# Phytophotodermatitis Due to Fig Tree Sap Activated by Ultraviolet Light: A Case Report

**DOI:** 10.7759/cureus.54286

**Published:** 2024-02-16

**Authors:** Maria Vilela, Sandra Fernandes, Ana Sofia Barroso, Ana Carvoeiro

**Affiliations:** 1 Internal Medicine, Unidade Local de Saúde do Alto Minho (ULSAM) - Hospital de Santa Luzia, Viana do Castelo, PRT; 2 Epidemiology and Public Health, Aces Cávado II - Gerês/Cabreira, Ferreiros, PRT; 3 Internal Medicine, Centro Hospitalar do Médio Ave, Santo Tirso, PRT

**Keywords:** light sensitizing, reaction by plants, painful rash, furanocoumarins, phytophotodermatitis

## Abstract

Phytophotodermatitis is a dermatological reaction caused by exposure to certain plants, which becomes activated upon subsequent exposure to sunlight. This can frequently result in a rash. Typically, supportive treatment is recommended. In this report, we describe the case of phytophotodermatitis in a 57-year-old man who experienced a painful rash with streaked lesions following the pruning of a fig tree during the summer. The patient, with no significant medical history, presented to the emergency department in July with a painful, streaked rash on both forearms. The lesions appeared overnight, predominantly on areas of skin exposed while sleeping. The patient denied contact with potential irritants and had not engaged in recent travel or altered his usual habits. Laboratory tests, including complete blood count and markers of inflammation, showed no abnormalities. A thorough patient history revealed recent fig tree pruning, a task usually undertaken in winter. The diagnosis of phytophotodermatitis was made based on the characteristic skin lesions and the patient's history of exposure to fig tree sap. Treatment with antihistamines led to improvement in symptoms, and the patient was discharged with a week-long course of antihistamines and advice to avoid sunlight and contact with fig trees. This case underscores the importance of a detailed medical history, especially in the context of dermatological lesions, to accurately diagnose and treat conditions like phytophotodermatitis.

## Introduction

Phytophotodermatitis (PPD) is an inflammatory skin reaction triggered by plants. This condition arises when the skin comes into contact with furanocoumarins, which become reactive under ultraviolet (UV) radiation, specifically UV-A [1,2]. PPD commonly presents as an asymmetric, non-itchy, but painful rash or erythema [1,3,4]. Clinicians diagnose PPD by identifying a history of exposure to furanocoumarins. Its prevalence remains undetermined, but occurrences are more frequent during warmer months. This is due to higher furanocoumarin concentrations in plants and increased UV exposure. Treatment primarily involves supportive care, focusing on identifying and avoiding the causative agent. Generally, patients with PPD have a favorable prognosis [1,2,4]. The following case highlights the diagnostic process and management of a PPD patient presenting in an emergency department (ED) setting.

## Case presentation

**Figure 1:**
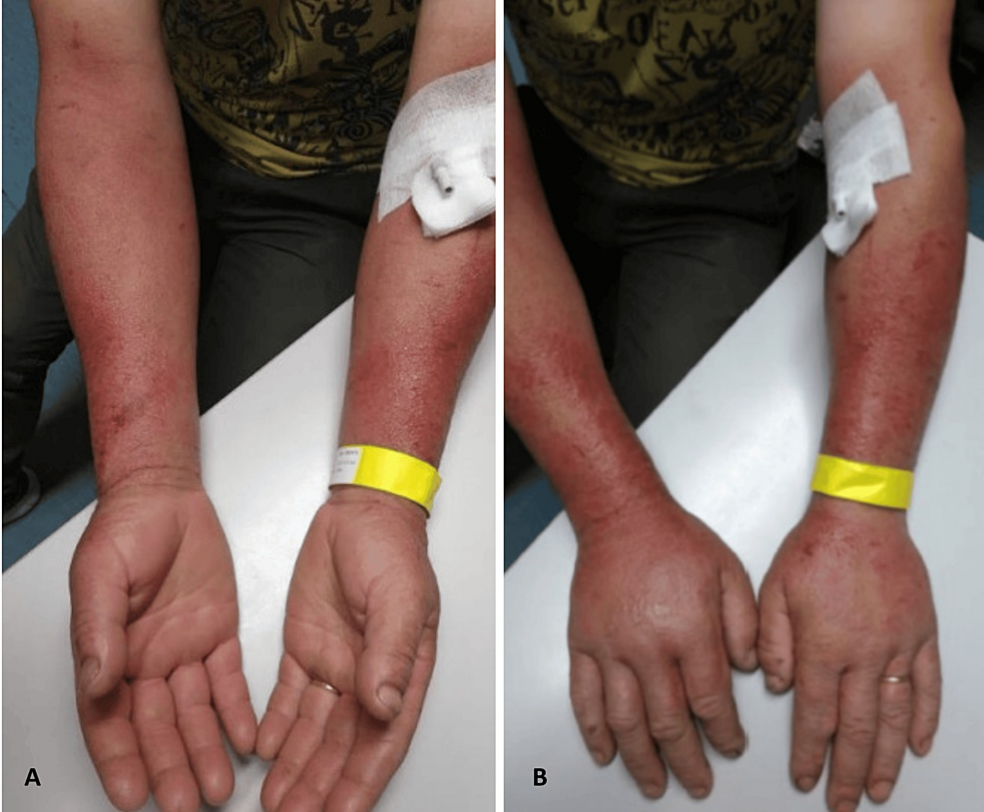
Supine (A) and pronated (B) arms showing the rash consequence of phytophotodermatitis

We report the case involving a 57-year-old man with no significant medical history. He presented to the ED in July with a painful rash on both forearms (Figure 1).

The rash emerged overnight, predominantly in areas of skin exposure, and featured streaked, thin lesions on both arms. These symptoms intensified progressively. The patient negated any recent contact with abrasive or cleaning substances, dietary alterations, new personal care products or detergents, interactions with animals, recent travel, self-inflicted harm, or similar past episodes. Upon detailed inquiry, the patient recalled pruning a fig tree in his backyard the previous day, a task he typically performed during winter.

Initial ED evaluations, including complete blood count, C-reactive protein, creatinine, and urea tests, revealed no abnormalities. A targeted review of literature in the ED suggested that the patient's symptoms were consistent with PPD, likely caused by sap from the fig tree. The patient received antihistamine treatment. After several hours under observation in the ED, he showed symptom improvement, notably pain relief and reduced edema. He was discharged with a seven-day prescription for antihistamines, instructions to avoid sunlight and fig trees in the near term, and advice to consult his family physician for follow-up.

## Discussion

Furanocoumarins, botanical substances found in certain plants like lime, celery, and fig trees, react to UV radiation through psoralen. This reaction typically results in a non-itchy erythema (skin redness) and operates independent of the immune system [1,4,5]. Symptoms usually appear 24 hours after exposure to furanocoumarins activated by UV-A radiation and can persist for weeks. Factors such as sweating, wet skin, and intense heat can exacerbate the condition [6]. PPD involves two distinct photochemical reactions: type I, which occurs without oxygen, and type II, which occurs in the presence of oxygen [2,5,6]. Both reactions disrupt cell membranes, leading to clinical manifestations such as erythema, blistering, epidermal necrosis, and skin peeling. Following the acute inflammatory phase, patients often experience post-inflammatory hyperpigmentation that can last for years [2,6].

The pattern of skin lesions in PPD varies depending on the mode of contact with furanocoumarins. For instance, drip-shaped lesions often appear on the arms after exposure to lime juice, while redness and swelling occur on exposed skin areas after consuming large quantities of furanocoumarin-rich foods [1,3,5,6]. For severe lesions, topical steroids may be beneficial. In rare instances, when lesions cover 30% or more of the total body surface area, hospitalization in a burn unit may be necessary for pain management and addressing potential skin lesions' potential complications [1,2].

The differential diagnosis includes a varied number of pathologies that cause skin lesions, including allergies and burns and in some cases autoimmune disorders [1,2,4,6]. 

Accurate diagnosis of PPD relies heavily on the patient's history, as it is primarily a clinical diagnosis. Often, the causative agent of the lesions is only identified through comprehensive patient history-taking (anamnesis). All laboratory data should support the diagnosis and help exclude other conditions in the differential diagnosis [2,4-6].

## Conclusions

Given that PPD is diagnosed clinically, anamnesis is crucial for identifying potential exposure to furanocoumarin-containing plants. In this case, the diagnosis was based on the characteristic presentation of a painful rash and bullae and the patient's history of exposure to a photosensitizing plant extract. Prevention is the most effective approach, and patient education is vital. This case underscores the significance of a thorough and focused anamnesis and highlights the necessity for vigilance, as routine activities can yield varied outcomes when performed at different times.
